# Seroepidemiological study of toxoplasmosis in women referred to a pre-marriage counseling center in Alborz Province, Iran

**DOI:** 10.1186/s13104-021-05581-0

**Published:** 2021-04-30

**Authors:** Melica Shahighi, Aliehsan Heidari, Hossein Keshavarz, Amir Bairami, Saeedeh Shojaee, Monireh Sezavar, Mahboobeh Salimi, Aref Teimouri

**Affiliations:** 1 Department of Medical Parasitology, School of Medicine, Alborz University of Medical Sciences, Karaj, Iran; 2 Department of Medical Parasitology and Mycology, Tehran University of Medical Sciences, Tehran, Iran; 3 Department of Experimental Sciences, Faculty of Allied Medicine, Alborz University of Medical Sciences, Karaj, Iran; 4 Department of Parasitology and Mycology, School of Medicine, Shiraz University of Medical Sciences, Shiraz, Iran

**Keywords:** Toxoplasmosis, Women, IgG avidity, ELISA, PCR, Iran

## Abstract

**Objectives:**

The aim of the current study was to assess prevalence of *Toxoplasma* infection and its associated risk factors in women of childbearing-age in central Iran.

**Results:**

Of 400 serum samples assessed for anti-*T. gondii* antibodies, 81 (20.25%) samples were positive for anti-*T. gondii* antibodies, including 74 positive samples (91.3%) for anti-*T. gondii* IgG and seven positive samples (8.7%) for IgG and IgM. Of seven IgG and IgM positive samples, five and two samples were high and low in IgG avidity, respectively. Based on PCR analysis, *Toxoplasma* infection was detected in one sample with anti-*T. gondii* IgM and low IgG avidity. The Chi-square test showed significant correlations of *T. gondii* seropositivity with history of undercooked meat consumption and contacts with cats (*p* < 0.05). In the present study, 79.75% of the participants were negative for IgG against *T. gondii* infection. Furthermore, recently acquired *Toxoplasma* infection was found using IgG avidity and PCR assays among women of childbearing-age in the study area, which would increase the risk of their fetus becoming infected. Educational program and antenatal screening of childbearing-age women for *T. gondii* infection may be important primary prevention strategies and help reduce the risk of congenital toxoplasmosis in this population.

## Introduction

*Toxoplasma gondii* (*T. gondii*) is an obligate intracellular protozoan that can infect warm-blooded vertebrates such as mammals and birds. In the life-cycle of *T. gondii*, domestic cats and felids as a definitive host harboring the sexual parasitic cycle and spreading oocysts through feces. They can infect a wide range of intermediate hosts including humans, birds, and other mammals, when ingested with food or water [1–3]. Detection of *Toxoplasma* immunoglobulin M (Toxo IgM) is the most common method used to assess the acute infection during pregnancy [4]. Toxo IgM usually reach detectable levels in the blood, nearly one week after the infection. However, discrimination between past and recent infections is a big challenge because Toxo IgM can persist for several months or years following the primary infection. Hence, diagnosis of acute *Toxoplasma* infections is not based on the measurement of IgM levels solely [4]. Recently, IgG avidity test is used to differentiate between acute and chronic infection. Avidity is known as the aggregate potency that a combination of polyclonal IgG antibody molecules bonds to antigen. The more duration of infection occurs, the stronger bonds are considerable [4–6]. In Iran, the overall prevalence of toxoplasmosis is 18–68% in various age groups of various regions of the country [7]. Primary infections in pregnant mothers can lead to protozoan transmission to the fetus, which results in increased risks of spontaneous abortions, severe congenital malformations and various disorders such as hydrocephaly and microcephaly [8–10]. Awareness of the *T. gondii* infection statuses in women referred to pre-marriage counseling centers can be an important indicator for estimating number of women at risk of toxoplasmosis during pregnancy [11]. Furthermore, this can help develop appropriate preventive methods such as education in premarital hygiene by identifying susceptible women to *T. gondii* infection and thus prevent congenital toxoplasmosis. In Iran and several countries (except France and Austria) pre-pregnancy tests for toxoplasmosis are not routinely carried out [12]. Based on several studies, seroprevalence of *T. gondii* infection varies widely from 4.6 to 97.2% in childbearing-age women in various regions of Iran [13]. Although a study has been carried out on the seroprevalence of toxoplasmosis in pregnant women in Karaj City, Alborz Province [14], no studies have been carried out to assess the seroprevalence of the infection in women of reproductive age in this region. Primary infections in pregnant women poses the highest risk for fetal infections. Therefore, this cross-sectional study was carried out to assess *T. gondii* infection and its associated risk factors in childbearing-age women referring to counseling centers in Alborz Province, Iran.

## Main text

### Methods

#### Study design

**Fig. 1:**
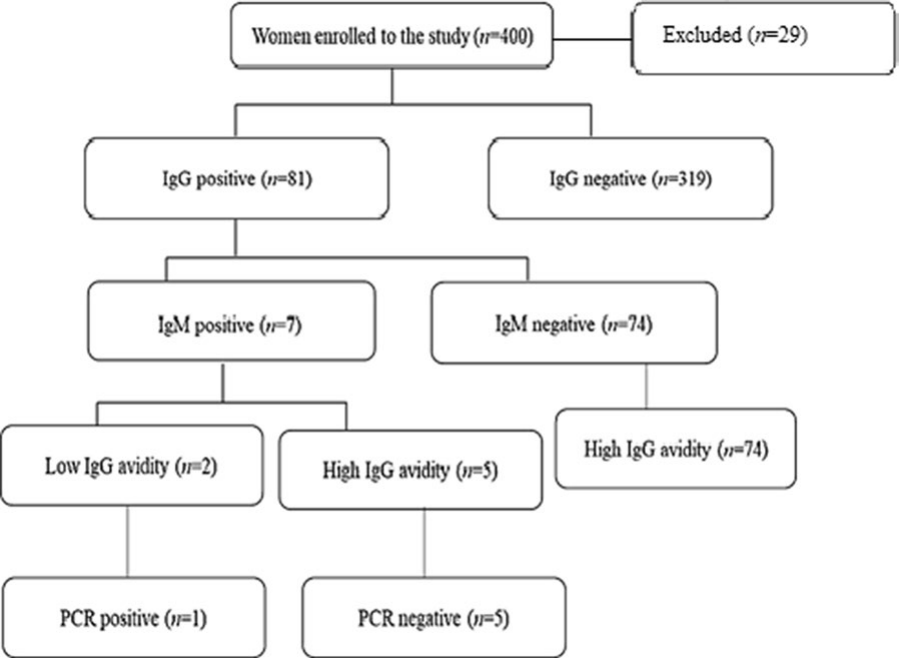
Flow chart for the selection of study participants, sample collections and data analyses

This descriptive-analytic study was a cross-sectional study, carried out in Alborz Province, from January to April 2017. Inclusion criteria for the participation of women included being married for the first time, not being pregnant, having no serious illnesses, being resident of Alborz Province, and willingness to participate in the study. The exclusion criteria included not being an Iranian, traveling to the province, or not being willing to participate in the study. In total, 29 women were excluded from the study (Fig. 1). The sample size was calculated based on the prevalence of toxoplasmosis in the region, using standard statistical formula (http://www.calculator.net/sample-size-calculator.html), given the prevalence rates of *T. gondii* as 29% [14], with a margin of error of 0.05, and a 95% confidence interval. This resulted in a sample size of 317. To take account of non-response rate the sample size was inflated by 30% to get a total sample size of 400.

#### Serum collection and assessment

Samples included 400 serum samples from women of childbearing-age aged between 15 to 45 years. A blood sample (up to 3.5 ml) was collected from each participant and blood serum was separated and stored at − 20 °C until use. The anti-*Toxoplasma* IgG levels of all collected sera were assessed using enzyme-linked immunosorbent assay (ELISA) and the IgG-positive samples were assessed for IgM. The IgG avidity test was carried out on IgG and IgM positive samples. Moreover, all IgM-positive blood samples were further assessed using polymerase chain reaction (PCR) method (Fig. 1). The specific assays used to test for IgG, IgM, IgG avidity, and PCR will be further explained below.

#### Risk factors

Through questionnaire survey of the participants, information was collected to assess risk factors at the time of sampling, as previously described [15].

#### Enzyme-linked immunosorbent assay

Detection of *T. gondii*-specific IgM and IgG was carried out using commercial Euroimmun ELISA kits (Euroimmun, Lubeck, Germany) based on the manufacturer's instructions [16].

#### Immunoglobulin-G avidity test

Avidity tests were carried out based on the standardized protocols originally described by Hedman et al. using Euroimmun kit according to the manufacturer’s instructions [17].

#### Genome extraction and polymerase chain reaction amplification

Genomic DNA was extracted from the blood samples with positive anti-*T. gondii* IgM using QIAquick PCR purification kit (Qiagen, Hilden, Germany) based on the manufacturer’s instructions. The *T. gondii* B1 gene was amplified based on a previously described protocol [18].

#### Statistical analysis

Analytical and descriptive statistics were carried out using IBM SPSS software v.21 (SPSS Inc., Chicago, IL, USA). Associations between the seroprevalence of *T. gondii* and the risk factors were reported using Chi-square test (bivariate test). The *p*-values < 0.05 were considered statistically significant.

### Results

#### Distribution of the participants

The participant average age was approximately 26.8 years (CI_95_ = 24.9–29.7). Participants were divided into six major age groups of ≤ 18, 19–23, 24–28, 29–33, 34– 38 and ≥ 39. A largest age group sampled was 29–33 (119/400; 29.75%).

#### Seroprevalence of Toxoplasma gondii-specific IgG and IgM antibodies

Of 400 serum samples assessed for anti-*T. gondii* antibodies, 81 samples (20.25%) were seropositive for anti-*T. gondii* antibodies and 319 samples (79.75%) were negative for Toxo IgG. Of the samples seropositive for anti-*T. gondii* antibodies, 74 samples (91.3%) positive for anti-*T. gondii* IgG and seven samples (8.7%) positive for IgG and IgM. Of the seven samples positive for anti-*T. gondii* IgM and IgG, five samples were high and two samples were low in IgG avidity assay (Fig. 1). One sample with Toxo IgM and low IgG avidity, analyzed using PCR and showed an expected size of 194 bp band for *T. gondii* B1 gene that was targeted in the PCR.

#### Associations of anti-Toxoplasma gondii antibodies with risk factors

Relative frequencies of anti-*T. gondii* IgG increased with increasing age (Table 1). As shown in Table 1, the highest relative frequencies within the age groups was seen in ≥ 39 years of age. Based on the findings presented in Table 2 and Chi-square test, significant associations were seen between the positive cases of *T. gondii* IgG and age of women (*p* = 0.008). Among the study participants, 1.25% (5/400) had cats in their homes, while 4.25% (17/400) frequently come in contact with cats because of neighbours owning cats. Chi-square tests showed significant correlations (*p* < 0.05) between *T. gondii* seropositivity and histories of undercooked meat consumption and contact with cats (Table 2).

**Table 1:** Frequency distributions of anti-*Toxoplasma gondii* IgG in 400 serum samples collected from childbearing-age women in special age groups using ELISA

Age group (year)	Positive serum no. (%)	Negative serum no. (%)	Relative frequencies within age groups (%)
≤ 18	1 (0.25)	26 (6.5)	1/27 (3.7)
19–23	9 (2.25)	71 (17.8)	9/80 (11.2)
24–28	20 (5)	70 (17.5)	20/90 (22.2)
29–33	31 (7.75)	88 (22)	31/119 (26)
34–38	12 (3)	47 (11.8)	12/59 (20.3)
≥ 39	8 (2)	17 (4.25)	8/25 (32)
Total	81 (20.25)	319 (79.75)	81/400 (20.25)

**Table 2:** Bivariate analysis of the risk factors associated with *Toxoplasma gondii* infection in 400 serum samples collected from childbearing-age women in Alborz Province, Iran

Variable	Positive serum no. (%)	Negative serum no. (%)	Odds ratio (95% CI) (bivariate analysis)	*p*-value
Consumption of raw/undercooked meat				
Yes	9 (2.25)	4 (1)	9.84 (2.94–32.8)	< 0.001
No	72 (18)	315 (78.75)		
Contact with cat				
Yes	8 (2)	14 (3.5)	2.55 (1.03–6.34)	0.04
No	69 (17.25)	309 (77.25)		
Occupation				
Housewife	49 (12.25)	181 (45.25)	1.74 (0.9–3.21)	0.76
Others^a^	32 (8)	138 (34.5)		
Education level				
Illiterate and primary	13 (3.25)	54 (13.5)	0.94 (0.48–1.82)	0.4
Diploma and university degree	68 (17)	265 (66.25)		
Age				
≥ 25 year-old	71 (17.75)	222 (55.5)	3.1 (1.53–6.27)	0.008
< 25 year-old	10 (2.5)			

^a^ Students, employees and unemployed individuals. Statistical analysis was carried out using chi-squared analysis. Significance was set at *p* < 0.05

### Discussion

Assessing women's immune statuses against toxoplasmosis before pregnancy can play an important role in preventing the fetus infection during pregnancy. In this study, 81 women (20.25%) of 400 women referred to the pre-marriage counseling center were diagnosed as positive for Toxo IgG. Previous studies on women of childbearing-age in various countries have shown prevalence of 58.7% in Krakow in Poland, 24.4% in Portugal, 48.3% in Cameroon, 33% in Venezuela, 81.4% in Ethiopia and 35.1% in Qatar [6, 19–23]. It seems that geographical conditions, dietary habits and spread of cats in each region are linked to differences between the results of this study and those of other studies [6, 14]. Climatic conditions affecting the survival of *T. gondii* oocysts in the environment and, hence, infection rates in meat-producing animals play a major role. Prevalence of *T. gondii* is high in humid tropical countries and conversely, low in arid and colder areas. However, anthropogenic factors explain a large part of the variations in human seroprevalence, including nutritional habits and hygiene practices in meat production [24]. In other studies on women of childbearing-age in Iran, prevalence of IgG anti-*T. gondii* was reported as 10.6% in Kerman Province, southern Iran [25] and 74.6% in Mazandaran Province, northern Iran [26]. Alborz Province is located in the foothills of the Alborz Mountains and has a climate with dry and hot sunny summers and relatively cold winters. Moreover, dietary habits including frequent consumption of undercooked meats, can contribute to the high infection level in Mazandaran Province than in Alborz Province.

In the present study, seven of the 400 total samples (1.75%) included IgM and IgG against *T. gondii*, which were lower prevalence, compared to 4% and 10.7% of women in childbearing-age with IgG and IgM in central Ethiopia and in the locality of Njinikom, North west of Cameroon, respectively [21, 23]. In Kerman, 2.3% of women of childbearing-age were positive for IgM against *T. gondii* [25]. This rate was 1% in women of this age in Portugal [20] and 1.43% in India [27], which were further similar to those from the current study. Based on the previous studies, IgM level against *T. gondii* could falsely remain high in blood for months or even years after exposure to the parasite [28, 29]. Furthermore, natural IgM might react with *Toxoplasma* antigens in absence of the infection [30, 31]. Therefore, an IgG avidity test was used to further differentiate between individuals with acute and chronic infections [32, 33]. In the current study, two of the seven sera with anti-*T. gondii* IgM showed low avidity, indicating possible acute infections. In a study on 128 pregnant women in Morocco, five women were positive for IgM against *T. gondii*, none of which showed low IgG avidity [34]. These findings are similar to findings from the present study and confirm that a positive current *T. gondii* IgM result is not necessarily diagnostic of an acute infection, since IgM can remain positive for several months or years following the primary *Toxoplasma* infection and false-positive IgM test results can occur. This is in overall agreement with previous reports of persistently positive IgM in chronic toxoplasmosis [28, 31, 35–37]. Therefore, some of the results are doubtful due to difficulty of accurately diagnosing of acute/chronic toxoplasmosis.

Prevalence of *T. gondii* infections in humans varies by age and seroprevalence usually increases with age in most regions, as shown in the current study [25, 27]. In the current study, significant correlations (*p* < 0.05) were found between the prevalence of *T. gondii* infection and history of exposure to cats and consumption of raw or undercooked meats (Table 2). These findings are similar to those from studies on women of childbearing-age in pre-marriage counseling centers in Kerman and Arak [25, 38]. In a study in Ethiopia, significant correlations were found between exposure to cats and rate of toxoplasmosis [21]. Conversely no correlations were reported in an Indian study between consumption of undercooked meat and toxoplasmosis, possibly because Indians rarely consume raw or undercooked meats [27]. In general, decreases in seroprevalence of *T. gondii* among women of childbearing-age increase proportions of pregnant women susceptible to primary infections, thus increasing the risk of congenital transmission. In contrast, in societies with high rates of *T. gondii* infection, most women are relatively immune to the infection during pregnancy and help reduce the risk of congenital toxoplasmosis because their previous exposures to the parasite before pregnancy.

### Conclusion

In the present study, 319 out of 400 (79.75%) serum samples from women were negative for IgG against *T. gondii*; therefore, these women were at risk of acquiring *Toxoplasma* infection during pre-marriage or pregnancy time. Moreover, recently acquired *Toxoplasma* infections were found using IgG avidity and PCR assays among women of childbearing-age in the study area, which would increase the risk of their fetus becoming infected. Health education approaches on toxoplasmosis and related risk factors and antenatal screening of childbearing-age women for *T. gondii* infection may be strategies for primary prevention of toxoplasmosis during pregnancy and help reduce the risk of congenital toxoplasmosis.

## Limitations

In this study, Toxo IgG was assessed using ELISA assay on all collected sera and then, Toxo IgM was assessed on positive samples. Toxo IgM assessment by ELISA in all collected sera was ideal, but was not possible due to financial constraints. This limitation for the study may have impacted the overall *T. gondii* IgM seroprevalence found in this study. Moreover, another limitation for the study include sampling bias, which only examines women who are about to be married and does not test women who are already married and are planning to have more children.

## Data Availability

All data generated or analyzed during this study are included in this published article. The raw data are available from the corresponding author on reasonable request.

## Abbreviations

Toxo IgM: *Toxoplasma* Immunoglobulin M
Toxo IgG: *Toxoplasma* Immunoglobulin G
ELISA: Enzyme-linked immunosorbent assay
IBM: International business machinescorporation
SPSS: Statistical package for the social sciences
PCR: Polymerase chain reaction
RAI: Relative avidity index
